# The Influence of Low-Molecular-Weight Monomers (TEGDMA, HDDMA, HEMA) on the Properties of Selected Matrices and Composites Based on Bis-GMA and UDMA

**DOI:** 10.3390/ma15072649

**Published:** 2022-04-04

**Authors:** Agata Szczesio-Wlodarczyk, Aleksander Polikowski, Michał Krasowski, Magdalena Fronczek, Jerzy Sokolowski, Kinga Bociong

**Affiliations:** 1 Laboratory of Materials Research, Medical University of Lodz, 92-213 Lodz, Poland; michal.krasowski@umed.lodz.pl; 2 Department of General Dentistry, Medical University of Lodz, 92-213 Lodz, Poland; aleksander.polikowski@stud.umed.lodz.pl (A.P.); jerzy.sokolowski@umed.lodz.pl (J.S.); kinga.bociong@umed.lodz.pl (K.B.); 3 “DynamoLab” Academic Laboratory of Movement and Human Physical Performance, Medical University of Lodz, 92-213 Lodz, Poland; magdalena.fronczek@umed.lodz.pl; 4 Department of Health Sciences, Medical University of Mazovia, 01-793 Warszawa, Poland

**Keywords:** bis-GMA, UDMA, TEGDMA, HDDMA, HEMA, dental resins, dental composites, mechanical properties, hardness, shrinkage stress

## Abstract

Bisphenol A-glycidyl methacrylate (bis-GMA) and urethane dimethacrylate (UDMA) are usually combined with low-viscosity monomers to obtain more desirable viscosity, handling characteristics and general properties. The present study determined the flexural strength (FS), flexural modulus (FM), diametral tensile strength (DTS), and hardness (HV) of five matrices and composites based on these resins. The polymerization shrinkage stress (PSS) was also studied for the composites. The polymer matrices were formed using bis-GMA and UDMA. TEGDMA, HEMA and HDDMA acted as co-monomers. The composites had 45 wt.% of filler content. The highest FS and FM were obtained from the UDMA/bis-GMA/TEGDMA/HEMA matrix and the composite (matrix + filler). The best DTS values were obtained from the UDMA/bis-GMA/HEMA matrix and the composite. One of the lowest values of FS, FM, and DTS was obtained from the UDMA/bis-GMA/HDDMA matrix and the composite. All the composites demonstrated similar hardness values. The lowest polymerization shrinkage stress was observed for the UDMA/bis-GMA/TEGDMA/HEMA composite, and the highest PSS was observed for the UDMA/bis-GMA/TEGDMA/HDDMA composite. The addition of HEMA had a positive effect on the properties of the tested materials, which may be related to the improved mobility of the bis-GMA and UDMA monomers.

## 1. Introduction

According to the Global Burden of Disease Study (2017), the most common health condition is untreated dental caries (tooth decay) in permanent teeth [1,2]. Its development is influenced by a range of genetic, physiological, environmental and behavioral factors. However, while this disease is largely preventable, its prevalence has barely reduced over the last 30 years [3]. 

The defective tooth tissue resulting from tooth decay is commonly repaired using dental composites. These materials are composed of resin matrix, fillers and additives (initiator and catalyst systems, and pigments). Since their invention in the late 1950s, dental composites have improved by means of better formulations and esthetic properties. However, there is still a need for further improvement in failure reduction and lifetime performance [3,4].

**Figure 1:**
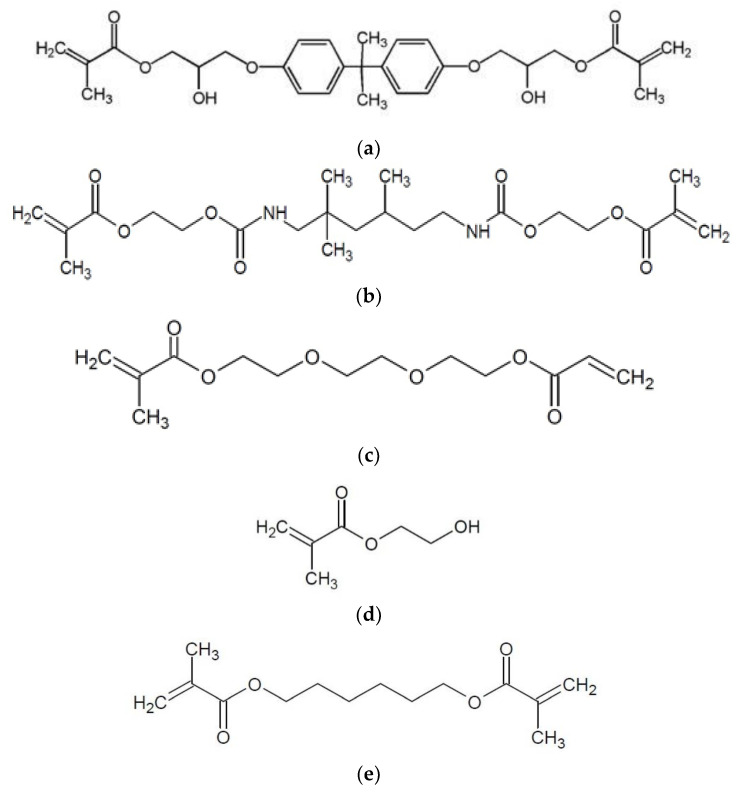
Chemical structures of bisphenol A-glycidyl methacrylate (bis-GMA, (**a**)), urethane dimethacrylate (UDMA, (**b**)), triethylene glycol dimethacrylate (TEGDMA, (**c**)), 2-hydroxyethyl methacrylate (HEMA, (**d**)) and 1,6-hexanediol dimethacrylate (HDDMA, (**e**)) monomers.

The most popular monomers used in polymer matrix formulations are bisphenol A-glycidyl methacrylate (bis-GMA) (Figure 1a) and urethane dimethacrylate (UDMA) (Figure 1b). To obtain proper viscosity and desirable properties, other methacrylate monomers are commonly used, such as triethylene glycol dimethacrylate (TEGDMA, molecular weight 286.32 g/mol) (Figure 1c), 2-hydroxyethyl methacrylate (HEMA, molecular weight 130.14 g/mol) (Figure 1d), 1,6-hexanediol dimethacrylate (HDDMA, molecular weight 254.32 g/mol) (Figure 1e), ethylene glycol dimethacrylate (EGDMA), ethylene diglycol dimethacrylate (DEGDMA), and 1,10-decanediol dimethacrylate (DDDMA or D3MA) [5]. The characteristics of individual monomers and interactions associated with resin formulation determine the properties of the polymer matrix [6].

Bis-GMA and UDMA are usually combined with a low-viscosity TEGDMA monomer, whose addition improves the degree of conversion, filler loadings and handling characteristics. However, it also decreases the general mechanical properties, and increases polymerization shrinkage and water sorption [7]. A common additive in dental adhesives is HEMA, which is characterized by small dimensions and polar properties (Figure 1d). HEMA reduces viscosity, and improves bond strength to dentin and co-monomer diffusion by expanding the demineralized collagen. This monomer also has the ability to improve the miscibility between monomers (hydrophilic and hydrophobic) and water [8,9,10]. HDDMA (Figure 1e) has a similar reactive group to bis-GMA; however, it is a relatively long and flexible molecule. Hence, it is often used as a cross-linking agent, and a functional monomer for polymers. HDDMA has low viscosity, good solvency and can be used as a diluent [11].

The aim of the study was to determine the influence of low-molecular-weight monomers (TEGDMA, HEMA, and HDDMA) on selected properties of the matrix and composites based on bis-GMA and UDMA. In particular, the effect of replacing the TEGDMA with HEMA or HDDMA monomers was investigated. The present study examined five matrices and composites based on these resins. The flexural strength, flexural modulus, diametral tensile strength and hardness were determined. The polymerization shrinkage stress was also studied for the composites. The null hypothesis was as follows: there is no effect of compositional changes in more complex resin systems on the FS, FM, DTS, HV and PSS of the materials. 

## 2. Materials and Methods

Five different resin mixtures were prepared according to the weight percentage of the selected monomers (Table 1). UDMA, bis-GMA, TEGDMA, HEMA and HDDMA were manufactured by Sigma-Aldrich (Merck, Kenilworth, NJ, USA). Each mixture contained camphorquinone (<1 wt.%) and N,N-dimethylaminoethyl methacrylate. The resins were also evaluated after being filled with 45 wt.% of silica (Arsil, Zakłady Chemiczne “RUDNIKI” S.A., Rudniki, Poland) and silanized with γ-methacryloxypropyltrimethoxy silane (Unisil Sp. z o. o., Tarnów, Poland). Filler (45 wt.%) was added to obtain good homogenization of ingredients while mixing with a mortar (the laboratory method of obtaining composites). 

The materials were cured for 20 s for 2 mm thickness using light-curing units (Mini L.E.D, Satelec, France). The lamp was characterized by a wavelength of 420–480 nm and a power of 1250 mW/cm^2^. The radiometer system (Digital Light Meter 200, Rolence Enterprice Inc., Taoyuan, Taiwan) was used to control the irradiance value. 

Flexural strength (FS) was established according to ISO 4049:2019 [12,13]. Six measurements were performed for each study group, based on rectangular samples (dimensions: 2 × 2 × 25 mm). The diametral tensile strength (DTS) of the dental materials was measured to evaluate their tensile properties [14,15]. DTS measurements were performed on cylindrical samples (diameter 6 mm and thickness 3 mm). Nine measurements were performed for each study group. The mechanical properties (FS and DTS) were determined using a Zwick Roell Z020 universal testing machine (Zwick–Roell, Ulm, Germany). The traverse speed was 1 mm/min in the FS test and 2 mm/min in the DTS test. Vickers hardness (HV) testing (Zwick ZHV2–m, Zwick–Roell, Ulm, Germany) was performed under a load of 1000 g for 10 s of dwell time. Nine measurements were performed per study group. The Zwick ZHV2–m hardness tester (Zwick–Roell, Ulm, Germany) was used for HV testing. All methods have been described previously [16].

The shrinkage stress (PSS) generated during photopolymerization of the composite materials was measured elasto-optically, as described previously [17,18]. In order to mimic an average tooth cavity, orifices (3 mm in diameter and 4 mm deep) were prepared in epoxy resin plates (Epidian 53, Organika-Sarzyna SA, Nowa Sarzyna, Poland). These plates were photo-elastically sensitive and had similar characteristics to dentin. The composite material was inserted into the orifices and cured for 20 s at the top and bottom using light-curing units (Mini L.E.D, Satelec, France). The generated strains in the plates were visualized using an FL200 circular transmission polariscope (Gunt, Hamburg, Germany). PSS was then calculated based on the modified Timoshenko equations [19]. Three samples were prepared for each composite material. 

A statistical analysis was performed with the use of Statistica 13 (Statsoft Polska Sp. z o. o., Krakow, Poland). The distribution of continuous variables was tested using the Shapiro–Wilk test of normality. Based on the results of the Shapiro–Wilk test, the Kruskal–Wallis test, with multiple comparisons of mean ranks, or one-way ANOVA, with post hoc test (Tukey’s HSD), were applied. The accepted level of significance was α = 0.05.

## 3. Results

### 3.1. Resin Materials

The results obtained during the evaluation of the polymer matrices are given in Table 2. The highest FS (92.9 MPa) and FM (1828 MPa) were obtained by UDMA/bis-GMA/TEGDMA/HEMA, while the lowest FS (73.4 MPa) and FM (1514 MPa) were demonstrated by UDMA/bis-GMA/HDDMA. The DTS ranged from 42.2 (UDMA/bis-GMA/HDDMA) to 64.3 MPa (UDMA/bis-GMA/HEMA), and the median HV ranged from 14 (UDMA/bis-GMA/HDDMA and UDMA/bis-GMA/TEGDMA/HEMA) to 16 (UDMA/bis-GMA/TEGDMA/HDDMA).

Statistically significant differences in FS were found for the following comparisons (Tukey’s post hoc test):

- UDMA/bis-GMA/TEGDMA/HEMA vs. UDMA/bis-GMA/HDDMA (*p*-value = 0.000130); UDMA/bis-GMA/TEGDMA/HDDMA (*p*-value = 0.0052); UDMA/bis-GMA/TEGDMA (*p*-value = 0.0007); UDMA/bis-GMA/HEMA (*p*-value = 0.0002);
- UDMA/bis-GMA/HDDMA vs. UDMA/bis-GMA/TEGDMA/HDDMA (*p*-value = 0.0187).

There are no statistically significant differences in the FM of the resin materials.

Statistically significant differences in DTS were found for the following comparisons (multiple comparisons of mean ranks for all groups):

- UDMA/bis-GMA/HEMA vs. UDMA/bis-GMA/HDDMA (*p*-value = 0.0015) and UDMA/bis-GMA/TEGDMA (*p*-value = 0.0179).

Statistically significant differences in HV were found for the following (multiple comparisons of mean ranks for all groups):

- UDMA/bis-GMA/TEGDMA/HDDMA vs. UDMA/bis-GMA/TEGDMA/HEMA (*p*-value = 0.0281).

### 3.2. Composite Materials

The FS, FM, DTS, HV and PSS for the composite materials are given in Table 3. The highest FS (95.7 MPa) and FM (4173 MPa) were obtained by the UDMA/bis-GMA/TEGDMA/HEMA composite, the lowest FS (79.3 MPa) by UDMA/bis-GMA/TEGDMA/HDDMA, and the lowest FM (3673 MPa) by UDMA/bis-GMA/HDDMA. The DTS ranged from 29.0 (UDMA/bis-GMA/HDDMA) to 37.4 MPa (UDMA/bis-GMA/HEMA). Similar hardness values were observed for all the tested materials. The lowest PSS was observed for the UDMA/bis-GMA/TEGDMA/HEMA composites and the highest for the UDMA/bis-GMA/TEGDMA/HDDMA blend. 

There are no statistically significant differences in the FS and PSS of the tested composites.

Statistically significant differences in FM were found for the following comparisons (Tukey’s post hoc tests for multiple comparisons):

- UDMA/bis-GMA/TEGDMA/HEMA vs. UDMA/bis-GMA/HDDMA (*p*-value = 0.0047), UDMA/bis-GMA/TEGDMA/HDDMA (*p*-value = 0.0348), and UDMA/bis-GMA/TEGDMA (*p*-value = 0.0070);
- UDMA/bis-GMA/HEMA vs. UDMA/bis-GMA/HDDMA (*p*-value = 0.0051), UDMA/bis-GMA/TEGDMA/HDDMA (*p*-value = 0.0380), and UDMA/bis-GMA/TEGDMA (*p*-value = 0.0077).

Statistically significant differences in DTS were found for the following (Tukey’s post hoc tests for multiple comparisons):

- UDMA/bis-GMA/HDDMA vs. UDMA/bis-GMA/HEMA (*p*-value = 0.0075).

Statistically significant differences in HV values were found for the following (multiple comparisons of mean ranks for all groups):

- UDMA/bis-GMA/TEGDMA vs. UDMA/bis-GMA/TEGDMA/HEMA (*p*-value = 0.0071).

## 4. Discussion

Due to their rapid evolution over the last two decades, dental composites are widely used as direct aesthetic tooth-resembling restorations [20]. Dental composites can be used successfully in small- to moderate-sized restorations, and can demonstrate adequate clinical performance in large restorations [21,22,23,24]. Unfortunately, despite continuous research, the ideal dental composite material remains elusive, and failures still occur, due to secondary caries and bulk/margin fracture of the dental composite. To improve composite performance, recent research has focused on the correct composition of the matrix and filler [20]. The present study evaluates the influence of low-molecular-weight monomers (TEGDMA, HDDMA, and HEMA) on the properties of the matrix and composite based on bis-GMA and UDMA, i.e., both as neat resins and composites with a 45 wt.% filler fraction. The null hypothesis can be rejected, due to changes in the evaluated properties, along with the modification of the resin’s composition. 

It is important to emphasize that the transfer of occlusal loads in restoration is complex. When compressive force is applied, tensile or even shear stresses can arise in the restoration material [25]. Therefore, to select the most suitable experimental material for use in composites, there needs to be a detailed evaluation of their mechanical and physical properties [26]. 

As the compressive strength of composite materials is much higher than their tensile strength (TS), and TS is more affected by internal flaws, this would appear to be the most suitable basis for a strength test for dental composites [27]. However, it is very difficult to perform tensile tests on brittle materials, such as composites. The following two methods are used to determine the ability of a brittle dental filling to resist tensile stress, which often occurs during chewing: FS test (ISO 4049) and DTS test (ADA No. 27). During an FS test, both tensile and compressive stresses occur in the sample. According to the ISO 4049 standard, in composite materials, the FS should be a minimum of 80 MPa for occlusal surface restoration and 50 MPa for other restorations [12]. Our data indicate that all the tested composites met the ISO 4049 standards for materials used in occlusal surface restorations (i.e., >80 MPa). 

The UDMA/bis-GMA/TEGDMA/HEMA matrix and composite materials demonstrated the highest FS and FM values. Statistically significant differences were observed in FS for the matrices and in FM for the composites (Table 2 and Table 3). Composite materials are more resistant to bulk fracture, cracking at the margins and wear, due to their high flexural strength [28,29,30]. Although the resins did not demonstrate a significantly higher FS compared to composite materials, significant differences were observed between the matrices and composites in the modulus values. The high FS values observed for the resins could be attributed to their elastic-plastic properties; the addition of filler, as in the composites, stiffens the system, increasing the elastic modulus, thus resulting in more brittle characteristics. 

The addition of the HEMA monomer had a positive effect on FS and FM, as also noted previously [31]. This beneficial phenomenon can be explained by the better mobility of bis-GMA and UDMA monomers in HEMA (6 mPa·s [8]); this may be due to the fact that HEMA has lower viscosity and better mobility than TEGDMA (10 mPa·s [32]). Previous studies have found UDMA/HEMA to demonstrate a higher degree of conversion than the unblended UDMA matrix [33]; the authors propose that the observed increase in DC results from the copolymerization of HEMA, as a monofunctional monomer, with UDMA, without participating in the reaction between pendant vinyl groups. This observation can also account for the fact that composites containing HEMA tend to have better properties. The HDDMA materials had similar properties to those containing TEGDMA. The addition of the HDDMA monomer resulted in a slight decrease in the modulus of elasticity, which is in agreement with previous findings [34]. As HDDMA has a low molecular weight (~252 g/mol) and a linear structure, it lowers the overall viscosity of the composite, allowing more filler or additional components to be incorporated. 

It has been found that an experimental composite consisting of bis-GMA, bis-EMA (40:40 wt.%) and HDDMA (20 wt.%), as an alternative to TEGDMA (20 wt.%), demonstrates comparable flexural strength (101.9 ± 5.5 MPa vs. 89.4 ± 5.7 MPa) to composites with a traditional matrix (bis-GMA/TEGDMA = 60:40 wt.%, FS = 101.6 ± 5.9 MPa) [35]. However, the HDDMA composite (partition coefficient LogP: 3.13) had significantly lower water sorption than the TEGDMA form (LogP: 2.42), resulting in the FS value being maintained at the same level after 30 days of water immersion [35].

The tensile strength test is difficult to perform for brittle materials, due to problems associated with sample preparation, alignment and gripping. Additionally, the obtained results may not be reliable [36,37]. In dentistry, the DTS has been proposed as an alternative method to determine the ability of a brittle dental material to resist tensile stress [13,38]. All the tested materials met American Dental Association Specification No. 27—Resin-Based Filling Materials, requiring a minimum DTS value of 24 MPa [39]. In fact, much higher DTS values were achieved for the matrices, i.e., without the filler. It should be emphasized that the DTS test assumes negligible deformation before fracture. Resin demonstrates greater plastic deformation than filled materials, which may result in some overestimation of the results [15,40]. The best values were achieved for the UDMA/bis-GMA/HEMA mixture, possibly due to the higher degree of conversion achieved, thanks to the better mobility of UDMA and bis-GMA monomers. It was shown that resin viscosity is an important determinant of the properties of resin composites [41].

Another commonly evaluated property of dental materials is hardness, which may be useful in the preselection of material composition [42]. Commercial dental composites demonstrate a very wide spectrum of Vickers hardness values, from around 30 HV to over 100 HV [43,44,45,46]. As restorative materials are intended to mimic tooth tissues, it is crucial that composites demonstrate hardness of at least 40–50 HV. The materials in the present study did not meet those criteria, due to having no filler content (resin) or only low levels (only 45 wt.%). This is consistent with other studies of experimental dental composites [16,47,48]. Composites demonstrate considerably greater hardness values (from 15 to 26 HV) than resins, due to the addition of filler. This increase is also affected by the type, morphology and size of the filler; increasing the filler content results in higher hardness [49,50]. In our present study, only 45 wt.% filler was added to obtain good homogenization of the ingredients while mixing with a mortar (the laboratory method of obtaining composites). 

Although correct selection of the filler type and amount should increase the level of hardness, to meet the requirements for dental materials, even materials with low hardness values can be used as lining materials or as restorative materials in class V and deciduous teeth. An example is the SDR composite (Dentsply); while it may have low DTS and HV values, it is covered with another material with better strength properties, which is exposed to occlusion loads (i.e., the chewing surface) [51].

One of the main reasons for restoration failure is secondary caries, which may develop due to the loss of marginal adaptation, the occurrence of leakage at the tooth/composite interface and the lack of antibacterial properties [52,53]. Therefore, polymerization shrinkage stress should be determined when evaluating dental composites. Such shrinkage at the tooth/restoration interface results in the development of stress, which may weaken the bond between the tooth tissue and composite material. The degree of shrinkage stress is influenced by a range of clinical conditions, including cavity preparation, curing method and, most importantly, material formulation [54]. The magnitude of contraction stress has also been found to depend on the curing characteristics and the photopolymerization rate of the dental resin composite [55,56].

In the present study, the shrinkage stress generated at the restoration interface was determined using the elasto-optic method. Shrinkage stresses for dental composites are known to range from around 1 to over 15 MPa [18,57,58,59]. It is worth emphasizing that the measurement method will also affect the determined values [54]. In our study, most materials showed similar stress values, these being around 10 MPa, and these values are comparable to those available on the market [60]. 

The shrinkage stress also depends on the amount of filler, which directly influences the resin content. A lower resin volume demonstrates less volumetric shrinkage and, hence, stress [53,61]. The tested experimental composites contained only 45 wt.% of filler, which is a small amount compared to commercial materials. Therefore, it can be assumed that the use of a higher filler content will not only increase the modulus of elasticity and hardness, but also reduce the shrinkage stress. One tested material, containing the four monomers UDMA, bis-GMA, TEGDMA and HEMA, showed low stress values, around 8 MPa. The visible difference in contraction stress observed between the UDMA/bis-GMA/TEGDMA/HDDMA (PSS = 10.4 ± 0.9 MPa) and UDMA/bis-GMA/TEGDMA/HEMA (PSS = 7.8 ± 0.6 MPa) composites can be explained by the lower polymerization reactivity of the HEMA monomer in comparison with the HDDMA monomer. Previous data indicate the following sequence of reactivity (high to low), based on induction times, viscosity changes and the modified Boltzmann sigmoidal model: UDMA > HDDMA > HEMA [62]. A lower polymerization rate could help in stress relaxation, as a result of improved macromolecule viscous flow or allowing the forming network to be rebuilt. By prolonging the increase in elastic modulus, it is possible to reduce and delay the growth of contraction stress; in such cases, it is still possible to deform the polymer chain and change its position (internal flow) [55,63].

Despite having similar FS and HV values, the presented composite materials with HEMA seem to have the most favorable properties, in terms of contraction stress, FM and DTS. In order to better understand the dependence between composition and physical properties, there is a need for a further degree of conversion analyses. 

## 5. Conclusions

All the tested composites based on bis-GMA/UDMA (40/40 wt.%) and low-molecular-weight monomers demonstrated satisfactory mechanical properties, i.e., flexural strength >80 MPa, diametral tensile strength >24 MPa, and hardness >25, making them a starting point for further research. However, the most promising are composites with a limited amount of HEMA. The addition of HEMA to the polymer matrix improves the mechanical properties, such as the hardness, diametral tensile strength, three-point bending strength and flexural modulus, of the composite. Additionally, the composite UDMA/bis-GMA/TEGDMA/HEMA (40/40/10/10 wt.%) showed the lowest shrinkage stress.

## Figures and Tables

**Table 1:** Composition of matrices. Composites used in this study have the same resin composition and contain 45 wt.% of silanized silica.

Matrix	Percentage of Individual Monomers (wt.%)				
	bis-GMA	UDMA	TEGDMA	HEMA	HDDMA
UDMA/bis-GMA/HDDMA	40	40	-	-	20
UDMA/bis-GMA/TEGDMA/HDDMA	40	40	10	-	10
UDMA/bis-GMA/TEGDMA	40	40	20	-	-
UDMA/bis-GMA/TEGDMA/HEMA	40	40	10	10	-
UDMA/bis-GMA/HEMA	40	40	-	20	-

UDMA—urethane dimethacrylate, bis-GMA—bisphenol A glycol dimethacrylate, TEGDMA—triethylene glycol dimethacrylate, HDDMA—1,6-hexanediol dimethacrylate, and HEMA—2-hydroxyethyl methacrylate.

**Table 2:** The results of flexural strength (FS), flexural modulus (FM), diametral tensile strength (DTS) and hardness (HV) for resin materials. Values are given as mean with standard deviation (SD) or median with quartile deviation (QT), based on distribution and homogeneity of variance. The results with the same assigned letter show a statistical difference at the level of *p* ≤ 0.05. The results without the assigned letter do not show a statistical difference.

Test	Result Type	UDMA/bis-GMA/HDDMA 40/40/20	UDMA/bis-GMA/TEGDMA/HDDMA 40/40/10/10	UDMA/bis-GMA/TEGDMA 40/40/20	UDMA/bis-GMA/TEGDMA/HEMA 40/40/10/10	UDMA/bis-GMA/HEMA 40/40/20
FS	M (SD)	73.4 ^a,e^ (7.2)	83.4 ^b,e^ (4.7)	81.2 ^c^ (2.6)	92.9 ^a,b,c,d^ (3.8)	79.2 ^d^ (2.7)
FM	M (SD)	1514 (250)	1603 (321)	1585 (241)	1828 (281)	1798 (139)
DTS	MD (QD)	42.2 ^a^ (9.6)	49.7 (4.8)	48.6 ^b^ (1.6)	55.5 (5.4)	64.3 ^a,b^ (3.7)
HV	MD (QD)	14 (1)	16 ^a^ (1)	15 (0)	14 ^a^ (0)	15 (1)

UDMA—urethane dimethacrylate, bis-GMA—bisphenol A glycol dimethacrylate, TEGDMA—triethylene glycol dimethacrylate, HDDMA—1,6-hexanediol dimethacrylate, and HEMA—2-hydroxyethyl methacrylate.

**Table 3:** The results of flexural strength (FS), flexural modulus (FM), diametral tensile strength (DTS), hardness (HV) and polymerization shrinkage stress (PSS) for composite materials. Values are given as mean with standard deviation (SD) or median with quartile deviation (QT), based on distribution and homogeneity of variance. The results with the same assigned letter show a statistical difference at the level of *p* ≤ 0.05. The results without the assigned letter do not show a statistical difference.

Test	Result Type	UDMA/bis-GMA/HDDMA 40/40/20	UDMA/bis-GMA/TEGDMA/HDDMA 40/40/10/10	UDMA/bis-GMA/TEGDMA 40/40/20	UDMA/bis-GMA/TEGDMA/HEMA 40/40/10/10	UDMA/bis-GMA/HEMA 40/40/20
FS	M (SD)	81.5 (10.9)	79.3 (11.1)	81.7 (5.0)	95.7 (10.5)	82.5 (13.4)
FM	M (SD)	3673 ^a,d^ (172)	3782 ^b,e^ (78)	3695 ^c,f^ (364)	4173 ^a,b,c^ (237)	4168 ^d,e,f,^ (122)
DTS	M (SD)	29.0 ^a^ (3.6)	31.8 (5.4)	33.6 (2.4)	32.8 (6.2)	37.4 ^a^ (5.8)
HV	MD (QD)	25 (1)	25 (0)	25 ^a^ (1)	26 ^a^ (1)	26 (1)
PSS	M (SD)	9.8 (1.6)	10.4 (0.9)	9.2 (0.9)	7.8 (0.6)	9.9 (0.1)

UDMA—urethane dimethacrylate, bis-GMA—bisphenol A glycol dimethacrylate, TEGDMA—triethylene glycol dimethacrylate, HDDMA—1,6-hexanediol dimethacrylate, and HEMA—2-hydroxyethyl methacrylate.

## Data Availability

Data available in a publicly-accessible repository: Zenodo. 10.5281/zenodo.6409794.
